# A New Possible Etiology in Wernicke Encephalopathy: A Clinical Case Report

**DOI:** 10.1192/j.eurpsy.2026.10883

**Published:** 2026-07-31

**Authors:** G. Eren, B. Koparal

**Affiliations:** Department of Psychiatry, Gazi University Faculty of Medicine, Ankara, Türkiye

## Abstract

**Introduction:**

Wernicke encephalopathy (WE) is a neuropsychiatric emergency caused by thiamine (vitamin B1) deficiency. Common etiologies include chronic alcohol use, malnutrition, and gastrointestinal surgery. Thiamine deficiency disrupts neuronal metabolism, leading to oxidative stress and cell death. This report describes a case of sudden-onset WE occurring after years of homemade alcohol consumption.

**Objectives:**

*This case emphasizes the importance of recognizing homemade or noncommercial alcohol consumption as a potential new etiological factor that could be considered for WE.* It highlights the neuropsychiatric impact of thiamine deficiency combined with toxic alcohol metabolites, underscoring the need for early diagnosis, high-dose parenteral thiamine, and multidisciplinary management for optimal recovery.

**Methods:**

Description of a clinical case and literature review of the current literature.

**Results:**

**Case Report**

A 58-year-old man with a 9-year history of daily alcohol intake presented with confusion, ataxia, and weakness. He had recently started consuming homemade alcohol of unknown composition. Physical examination revealed cachexia and erythematous dorsal hand lesions initially suggestive of pellagra.

Mental status examination showed a disoriented, withdrawn patient with psychomotor retardation and limited cooperation. Speech was slow, dysarthric, and occasionally incoherent. Mood was euthymic with blunted affect. Thought content lacked delusions or hallucinations, but family reported transient visual hallucinations during prior episodes. Insight and judgment were impaired.

Neurological findings included horizontal nystagmus, dysdiadochokinesia, ataxia, and hypoactive reflexes with negative Babinski. MRI demonstrated T2 hyperintensity in the mammillary bodies and cerebellar atrophy consistent with thiamine-deficiency encephalopathy.

**Clinical Course**

He was treated with intramuscular thiamine (600 mg/day), N-acetylcysteine (1200 mg/day), risperidone (1 mg/day), electrolyte replacement, and nutritional support. Within two months, mental clarity, ataxia and oral intake improved; hand lesions healed. At one-year follow-up, only mild ataxia remained; full cognitive recovery was observed.

**Image 1:**
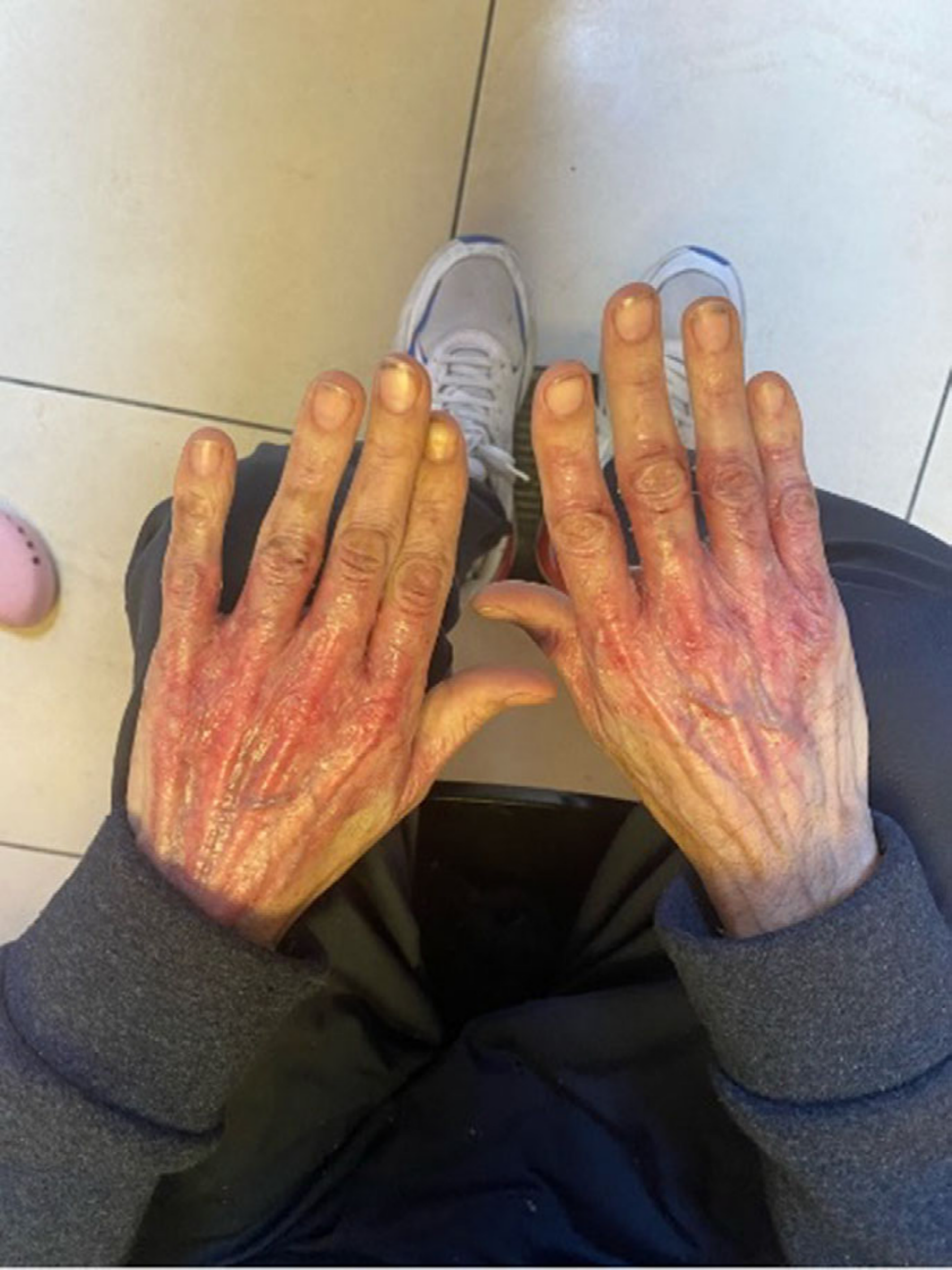

**Image 2:**
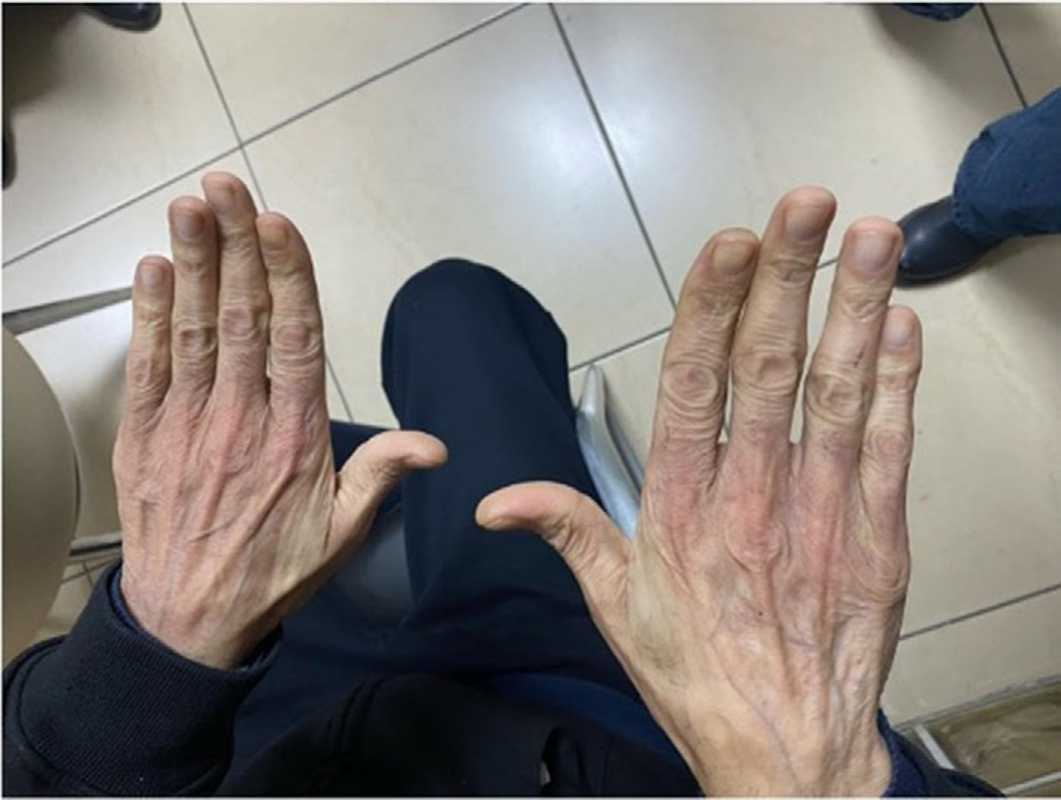

**Conclusions:**

Homemade alcohol combined with thiamine deficiency can exacerbate neuropsychiatric injury.

Early recognition and aggressive thiamine therapy reduce morbidity and mortality. Magnesium optimizes thiamine activity, and psychosocial support improves outcomes. WE should be managed as both a neurological and psychiatric emergency requiring multidisciplinary care.

*To our knowledge, this is the first case linking homemade alcohol use to the development of WE as a potential new etiology.*

**Disclosure of Interest:**

None Declared

